# Mindfully Reframing the Psychological Impact of the COVID-19 Outbreak Through a Social Media Community for Students: A Pragmatic Study

**DOI:** 10.3389/fpsyg.2021.566778

**Published:** 2021-06-24

**Authors:** Francesco Pagnini, Elisa Bonalda, Eleonora Montrasi, Elena Toselli, Alessandro Antonietti

**Affiliations:** ^1^Department of Psychology, Università Cattolica del Sacro Cuore, Milan, Italy; ^2^Department of Psychology, Harvard University, Cambridge, MA, United States

**Keywords:** COVID-19, coronavirus, psychological intervention, online community, stress, coping, cognitive flexibility, openness

## Abstract

The COVID-19 outbreak and the restrictions that have been enforced by the health authorities are having a profound psychological impact on the population. Many people, including the students, faced forced modifications to their daily lives and this prompted the need for scalable strategies to promote resilience. We designed an online community intervention for psychology students and recent alumni aimed to promote functional coping strategies through openness and cognitive flexibility. This psycho-educational intervention was delivered through a private group on social media (Facebook) during the acute phase of the lockdown period and it involved the publication of exercises and quick lectures. Contents were posted regularly and members of the community were invited to share their comments. The posts included stimuli that promote open and flexible reflections on the current situation. The overall aim of this group was a cognitive reframing on the epidemic effects, promoting creative and flexible thinking. We ran a thematic analysis of the interactions and we collected qualitative feedback at the end of the intervention. The participants' comments dealt with changes in their perspectives, sharing discomfort, encouragement and support, and building a sense of community. Post-intervention comments were highly satisfied and confirmed the helpfulness of the intervention to promote flexibility and openness, eventually helping to manage the negative emotions related to the COVID-19 outbreak. This study provides preliminary evidence that an online psycho-educational community stimulating flexibility and openness can help to reframe the negative psychological impact of the outbreak, improving their management.

## Introduction

The COVID-19 pandemic has spread around the world since December 2019, infecting millions of people and causing hundreds of thousands of deaths (World Health Organization., 2020a). In response to the outbreak, several countries have implemented lockdown and other movement restriction strategies to prevent the virus from spreading. Countries have taken unprecedented measures, which varied across nations. Italy, which has been one of the worst-hit countries by the pandemic (at least in the first phase outside China), imposed a strict lockdown for several weeks. Together with the medical, social, and economical implications of the epidemic, there are multiple psychological consequences (Pfefferbaum and North, 2020; World Health Organization., 2020b). Stress, anxiety, depressive symptoms, sleep problems, and fear have globally increased (Torales et al., 2020). For example, an analysis of social media contents after the outbreak showed an increase in anxiety and depression symptoms and a reduction of life satisfaction and positive emotions (Li et al., 2020).

The lockdown that was implemented by many health authorities in the world restricted personal and work activities, limiting movements, and social contacts. This often resulted in a sense of uncertainty and loss of perceived control, with a negative impact on the psychological well-being (Mertens et al., 2020).

A large survey conducted in Italy at the beginning of the outbreak suggested that cognitive flexibility may be a protective factor against the negative psychological consequences of the COVID-19 situation (Pagnini et al., 2020). This is in line with previous studies reporting that psychological flexibility is a fundamental aspect of well-being (Kashdan and Rottenberg, 2010). A flexible mindset allows an improved adaptation and it helps to cope with challenging situations. Another psychological characteristic that resulted associated with improved well-being and functional coping during the epidemic is openness (Bogg and Milad, 2020; Pagnini et al., 2020), which refers to receptivity to new experiences and ideas. Openness and flexibility are also core components of creativity, which requires to widen the perspectives, connect the dots, and re-organize the relationships (Colombo et al., 2018). The importance of these constructs for well-being has been reported multiple times in the literature. In particular, the Langerian approach to mindfulness defined as the process of making novel distinctions, which includes openness, flexibility, curiosity, and creativity. According to this model, a mindful reappraisal can be provided when one realizes that there are different perspectives on the same aspect (Pagnini and Langer, 2015). This is the essence of Langerian mindfulness and has been repeatedly associated with quality of life (Langer, 1989; Pagnini and Phillips, 2015). Therefore, an improvement of these psychological characteristics generally results in improved well-being and coping skills (Pagnini et al., 2016). Following this theorical idea, we developed a specific intervention aimed to reframe some negative perspectives about the pandemic and the social restrictions that were imposed during the first lockdown in Italy.

Social media played a particular role in disseminating the information about the outbreak, which sometimes included pitfalls such as fake news and panic transmission (Depoux et al., 2020; Salvi et al., 2021). Despite these risks, these forums can provide a learning space where self-efficacy and coping strategies can be built (Tower et al., 2015), which could represent a scalable and easily implementable strategy to promote adaptive coping against the COVID-19 situation (Van Bavel et al., 2020). During the first period of the pandemic, in Italy, social media were mainly used to exchange information and to share the common sentiment (Bhat et al., 2020), but they rarely provided structured interventions. A rapid review of the available literature on stress interventions for health care providers dealing with coronavirus (Callus et al., 2020) only identified one study protocol (Azam et al., 2019) using a digital intervention. More in general, the worldwide outbreak introduced new social and psychological challenges, which have been faced with both traditional psychological strategies with context-adjusted solutions, such as online consultations (Swartz, 2020), and innovative approaches, such as using VR or other technologies (Riva et al., 2020). Other studies explored the impact of online psychotherapy as a way to reduce coronavirus-related worries (Wahlund et al., 2021) or other forms of anxiety (Chen, 2020).

The project aimed to deliver, through a dedicated Facebook group, a group intervention that lead to a mindful reframing of the current situation. In other words, the goal of this intervention was to boost psychological flexibility, openness, and creativity through an active psycho-educational intervention. Group members were invited, with different stimuli, to embrace an open perspective about the challenges faced during the lockdown. The goal of the group was not only to ease the negative impact, but also to see the potential for growth and development. In this pilot study, we invited psychology students and recent alumni to join the group and its active discussion. The interactions and the subjectively reported effects were qualitatively assessed.

## Methods

### Design

**Figure 1:**
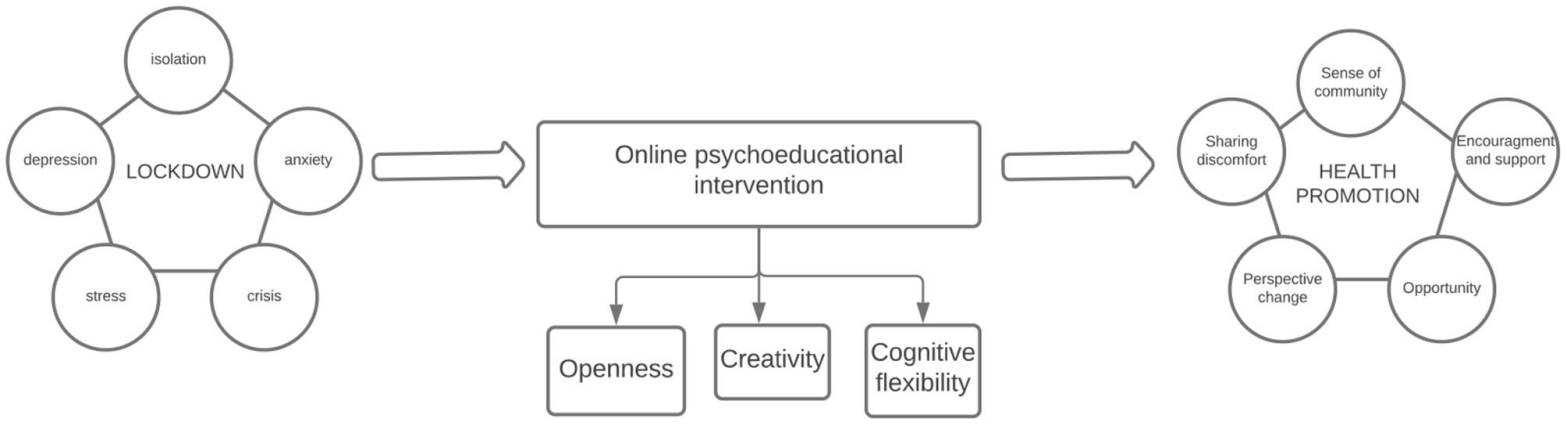
Study procedure.

We conducted a pilot study to explore the interactions and the subjectively reported effects of a Facebook group, designed to stimulate cognitive flexibility, openness, and creativity, through stimuli and discussions. This research is an “active analysis” on Facebook (Eysenbach and Till, 2001), as there was an active interaction between research members and the participants (Franz et al., 2019). After the end of the intervention, the group members were invited to express their views on the program (see Figure 1).

### Intervention

On March 20, 2020, we created a private group on Facebook, titled “Coronavirus and quarantine… Shall we grow together?.” About every day or any other day, a stimulus was posted on the discussion wall. These stimuli were either reflections to boost flexible thinking, which included sentences aimed to reframe the perspective on the current situation (e.g., “What will you miss of the lockdown, when it will be over?”) or exercises, which suggested activities that prompted a different perspective, such as brushing teeth with the non-dominant hand or to search for unexpected details in a movie. One of these exercises consisted of the voluntary sharing of a personal problem, with the invite to the other group members to help reframing or coping. Contents were all accompanied by an image to facilitate their visibility and improve the engagement. The group was moderated by a member of the research team (EB) and participants were able to reply to the existing posts, but not to create new posts on their own. All the contents were posted in Italian.

### Participants

We invited undergraduates, master's students, and recent alumni (>3 years) from the Faculty of Psychology at Università Cattolica del Sacro Cuore (UCSC) in Milan, Italy, to join the group. A direct invitation was sent to their university-provided email address and announcements advertising the group were posted in UCSC students' dedicated groups and pages. A total of 436 people joined the group, out of about 2,500 students and alumni contacted. When they joined the group, they accepted the posting instructions, which also included the consent for analyzing group data. The study was approved by the Ethics Commission of the Department of Psychology at UCSC.

### Data Collection and Analysis

All comments and reactions that were produced by the participants were collected over the 5-week period in which the intervention was conducted and put in a textual database through a careful copy-and-paste process. We recorded all the comments, the de-identified person who made them, and the number of “likes” and “reactions” (i.e., emotional reactions: Love, Fun, Wow, Sad, and Angry). Comments were examined with a thematic analysis (Braun and Clarke, 2006). This approach offers a robust and sophisticated toolkit for the analysis of qualitative data, is mostly appropriate when there is no deep theoretical commitments, and allows to explore the patterns across data (Braun and Clarke, 2014). Two researchers (EM and ET) independently immersed themselves in the materials, assigning the preliminary codes and extrapolating the categories. Once independently created the categories, the researchers reviewed them with a third researcher (FP) and reached a consensus on the identified themes.

After about a month since the last post, a request for feedback was posted. In this message, there was an invitation to complete a short survey on the program satisfaction, implemented with the Qualtrics suite (Qualtrics, Provo, UT). The survey included a general evaluation on the group helpfulness on a Likert scale (1 = very helpful; 2 = helpful; 3 = quite helpful; 4 = not very helpful; and 5 = not helpful at all) and some open-ended questions on the use of the stimuli and the discussion, including pros and cons. Specifically, the open-ended questions were: “How did exercises and reflections impact your life”?; “How was this group helpful, if that was the case?”; “What were the aspects of the group that you mostly appreciated?”; “What were the aspects that you did not appreciate?”; and “What would you recommend we change, should we repeat this experience?.” The answers were assessed with another thematic analysis, following the same procedure previously described.

## Results

### Group Activities and Participants

In the 5-week period of the intervention, there were a total of 28 posts, plus those dedicated to welcome the participants and asking to complete the final comments. Overall, there were 100 comments to the contents, made by 42 different participants, and 697 reactions. Of the 436 participants, 384 (88%) were female, with 266 (61%) ranging between 18 and 24 years, with 133 (30.5%) with an age between 25 and 34 years, and 37 people (8.5%) aged 35 or older.

### Analysis of the Interactions

The thematic analysis of the comments to the posts provided four key categories: “perspective changes to face the limitations,” “sharing discomfort,” “encouragement and support,” and “building a sense of community” (see Table 1). Participants' IDs are reported in brackets.

**Table 1:** Categories from the analysis of the interactions.

**Category**	**Definition**	**Example**
***Perspective changes to face the limitations***	Reframing the meaning of the lockdown: from crisis to opportunity	“*I played board games with my family*” (#13)
***Sharing discomfort***	Expression of difficulties and negative emotions	“*I miss physical contacts with my loved ones*.” *(#22)*
***Encouragement and support***	Empathy and emotional support towards others' comments	“*I feel very close to you*” (#4)
***Building a sense of community***	Feeling of belonging and social recognition	“*We are all in the same boat*” (#13)

*Italics represent verbatim from the participants*.

#### Perspective Changes to Face the Limitations

Comments included in this category referred to the shared strategies and to the attempts to change the perspective on the negative aspects of the lockdown. These contents explained how the students managed the restrictions of Covid-19 and their personal ways to adapt to change. The focus on the positive side of the condition emerged because they cognitively reframed the bad valence of isolation at home. In detail, the focus of attention switched from complaining about what it was not permitted to appreciating what they could do from home: “*I learned to do yoga*” (#2); “*I learned how to cook*” (#5); “*I spent time sunbathing on my balcony*” (#8). Many participants became aware of the importance to take a break from their previous busy life and enjoy more quality time with themselves and their family members (e.g., “*I played board games with my family*,” #13). Furthermore, several students stated that they often had video calls with friends and relatives whom they have been distant for long, as they would not generally meet in person.

#### Sharing Discomfort

This category includes comments concerning difficulties and negative emotions experienced by the students during the quarantine. Many comments from this theme refer to nostalgic feelings for social proximity and emotional closeness: “*I miss physical contacts with my loved ones, which is a language itself* ” (#22) and “*I miss hugging my grandparents*” (#36). Moreover, some members expressed a feeling of confusion and uncertainty, as “*I feel very sad and empty. The worst feeling is the constant perception of disorientation about my future. I get up every morning with no goals*” (#34) or “*In particular, all this uncertainty upsets me*” (#38).

#### Encouragement and Support

The most common reactions in response to the shared discomfort were encouragement and support. Sometimes participants showed empathy and emotional support: “*I feel your difficulty*” (#33), “*I totally understand you, I feel very close to you*” (#4), “*You are not alone*” (#2), or “*My friends and I are facing similar problems*” (#21). Sometimes people gave practical advices: “*I created a game with my nephews that consists of sharing nice words to communicate how much we miss each other!*” (#29), or “*I write a thought on my diary every night. It is a good way not to forget what the present is teaching us*” (#20).

#### Building a Sense of Community

The final category is populated by comments referring to the idea that students were building a sense of community and a form of social recognition: “*The first reason why we should feel closer is our common destiny: we are all in this together, we are facing the same restrictions, feeling the same fears and wishes*” (#24) or “*We are all in the same boat*” (#13). It is noteworthy to highlight that a great number of comments included both encouragement and a sense of community: “*It is in difficult times that we bring out the best in us!*” (#18), “*Most of us are struggling with this new situation because we are used to exerting control over events. However, the most important thing is adapting to a new routine, not to be pessimistic and appreciate simple things*” (#10), or “*Our mind is struggling because of anxiety and worry, but we shouldn't let this thing dividing us. What unites us is a sense of responsibility toward each other. For the first time, most of us are called to act not only for one's own sake, but especially for other's*” (#5). Another student said that “*The goal is not to sow panic by pointing out to each other, but to provide ideas and resources. One day I need you and the day after you need me, and I do anything possible to give you a starting point. This is beautiful*” (#32). It was also reported that feeling part of a large community while dealing with such a heavy catastrophe plays a significant role to fight loneliness.

### Post-intervention Survey

**Figure 2:**
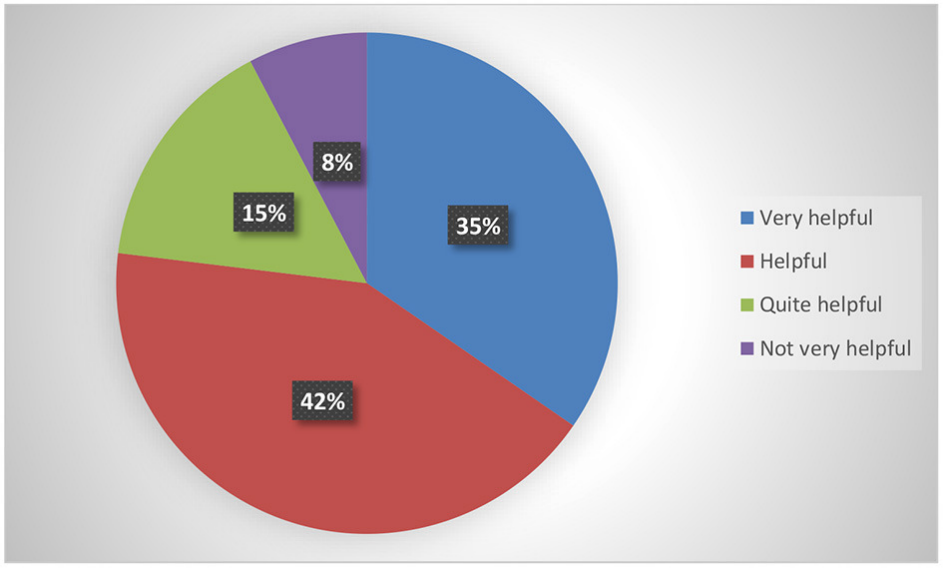
Overall satisfaction with the group.

The post-intervention survey was completed by 25 participants. This self-selected sample was composed of 21 females (84%) and 4 males (6%), with an average age of 24.87 years. Of these, 6 (24%) declared to have commented at least one post, 12 (48%) reported that they interacted with “likes” or other reactions, and 7 (28%) only read the stimuli. Overall, 9 (35%) considered the program very helpful, 11 (42%) helpful, 4 (15%) quite helpful, and 2 (8%) not very helpful (see Figure 2).

Both exercises and stimuli were particularly appreciated, as they prompted an open perspective on the lockdown: “*They made me see a new point of view on the coronavirus-related limitations*” (#9) or “*The group helped me to open up my mind*” (#18). Some students reported that the contents allowed them to manage their emotions: “*They toned down the perceived magnitude of certain events that seems hard to manage*” (#14) or “*It was helpful to see new opportunities and it allowed me to improve the management of my feelings*” (#17). Sometimes, participants shared the exercises and the reflections with other people outside of the group: “*I shared them with my relatives*” (#12), “*I used the exercises alone, but sometimes I discussed them with others, which made them more fun*” (#25). Together with the contents (e.g., “*Reflections and exercises have been very stimulating*,” #20), the group and the interactions have been particularly appreciated: “*I liked some original replies from the other group members*” (#9) or “*I appreciated the opportunity of supporting each other*” (#14). The limits that have been reported by the group members were focused on the limited ways of interaction (“*Sometimes, the discussion was limited*,” #6) and the fact that some contents were perceived as slightly repetitive (“*After a little while, contents got a little repetitive*,” #23). Finally, some suggested that the frequency of post publication was too intense (“*Maybe the contents were too frequent*,” #3).

## Discussion

As a response to the psychological threat represented by the COVID-19 outbreak and its social implications, we developed a structured psycho-educational program, delivered through a private Facebook group for psychology students, to enhance cognitive flexibility, creativity, and openness. Once analyzed the interactions and the post-intervention feedback, it seems that these qualities have been positively impacted, at least for those who actively joined the discussion and provided feedback. The largest theme identified, in fact, is plenty of sentences that suggest that flexible and open new strategies have been proposed and adopted. This is also confirmed by the answers to the post-intervention questions. New perspectives and flexible thinking, as well as openness and creativity, seem to be the most referred problem-solving strategies. In particular, most stimuli aimed to help to reframe the challenges of the COVID-19-related situation, trying to see positive aspects and growing opportunities out of the lockdown and social restriction norms. These strategies were reported as successful in managing negative emotions and in promoting well-being, in line with previous findings from the literature (Kashdan and Rottenberg, 2010). In comparison with existing online psychological interventions (e.g., Chen, 2020; Wahlund et al., 2021), participating in this social community requires a limited time engagement, which may increase its generalizability.

Thinking flexibly and positively about these challenges does not mean to deny the stressful and perhaps depressive aspects of the outbreak, as already identified by other studies (Saita et al., 2021). Participants expressed their need to share emotions, in the search for recognition and acceptance. The reactions to these disclosures were highly accepting and supportive. Together with some creative strategies to face adversities, the community responded with encouragement and recognition. The sense of community theme exemplifies the effects of these interactions, but it also describes the feeling that the whole human community has been affected. In line with this, it is worth noticing that some participants decided to practice the exercises and to share reflections with relatives and friends. In a sense, the slogan “We are all in this situation together” summarizes the feelings of connectedness, which may have grown despite the need for social distance (Ahmad et al., 2020). This pilot study supports the idea that the promotion of a mindful attitude, despite the psychological challenges posed by the outbreak and its social implications, can foster psychological well-being. This is in line with previous studies about interventions aimed to promote flexibility, openness, and mindful reframing (Garland et al., 2015). To our knowledge, this is the first work promoting this approach through social media and the first using it to face the psychological challenges of the COVID-19 pandemic.

Despite the coherence of these results and the clear narrative that arises from their interpretation, the study includes several limits. First of all, a comparison between the participants' level of wellbeing pre- to post-intervention was not possible, since we did not measure any related construct neither before nor after the study took place. We only have data from those who interacted and replied to our stimuli and questions. We know nothing about the impact of the intervention on those who did not interact. We suggest that at least some of those who did not comment may be positively impacted by the training, for two reasons: Among those who responded to the final survey, the ones that reported no interaction expressed a positive view on the utility of the program; secondly, we have anecdotical evidence that some students, who did not engage group discussions, have found it helpful. However, it is hard to quantify the actual impact from the available data. Thirdly, generalization is problematic: The study was conducted in Italy, with students and alumni from a private academic institution, and focused on online data. Moreover, the obtained results refer to a self-selected group of psychology students and the responders to the survey were few, preventing more extensive quantitative analyses. Finally, to keep short the post-intervention survey, we did not collect many details about demographics and individual characteristics of the responders. Further data on different populations are therefore warranted.

## Conclusions

The study provides some insights on the potential for an intervention that promotes flexible thinking, openness, and creativity, as a way to deal with the outbreak and the lockdown. The intervention, in this case, is delivered through a widely used social media, which makes it extremely scalable and cost-effective. It requires the creation of a community, by using a private group, allowing participants to reply to each other, easing recognition and encouragement. Future studies should explore, in the context of the lockdown or other challenging situations, the effects of the intervention with a controlled study and with a quantitative approach.

## Data Availability Statement

The raw data supporting the conclusions of this article will be made available by the authors, without undue reservation.

## Ethics Statement

The study was reviewed and approved by Ethics Commission of the Department of Psychology at UCSC. The participants provided their written informed consent to participate in this study.

## Author Contributions

FP and AA designed the study and the intervention. EB managed the intervention, in collaboration with FP and AA. EM and ET, in collaboration with FP, conducted the analyses. FP drafted the manuscript and all authors integrated the text with additions and comments. All authors approved the final version of the manuscript.

## Conflict of Interest

The authors declare that the research was conducted in the absence of any commercial or financial relationships that could be construed as a potential conflict of interest.
